# Supplementary material for: Differential Consequences of Bmp9 Deletion on Sinusoidal Endothelial Cell Differentiation and Liver Fibrosis in 129/Ola and C57BL/6 Mice
Source: Cells. 2019 Sep 13;8(9):1079. doi: 10.3390/cells8091079 (PMC6770219; doi:10.3390/cells8091079)
Supplement: Supplementary file 1 [file cells-08-01079-s001.pdf]

Supporting Information Table1. List of Primers

Primers for quantitative RT-PCR were designed using Primer-Blast on GenBank sequences and are separated by at least one intron or span an exon-exon junction for intron containing genes.

Gene	GenBank sequences	Forward (5'-3')	Reverse (5'-3')
Rpl13a	NM_009438.5	CCCTCCACCCTATGACAAGA	TTCTCCTCCAGAGTGGCTGT
Stab1	NM_138672.2	TCACTGTCCCCACACTACTTT	TGTCGCAACGTTTAGACCGTA
Stab2	NM_138673.2	CACTATGTCGGGGATGGACG	GGGAGCGTAGGTGGAATACG
Plvap	NM_032398.2	AGCACACTGCCTTCTCCTTG	AGCACACTGCCTTCTCCTTG
Nos3/eNos	NM_008713.4	CCAGCACCGGAGCCTAGC	AGGGTGTCGTAGGTGATGC
Cd209b	NM_026972.5	TGGGCTCCTGCTGATCATT	TTCCCTTGGGAGATGGGGAT
Ehd3	NM_020578.3	CGCCGTGCTTGAAAGTATCAG	ATAATTCGGTCCACCCGCTC
Maf	NM_001025577.2	AGGATGGCTTCAGAACTGGC	GGTCTCCACCGGTTCCTTTT
Gata4	NM_008092.4	ACCCTGGAAGACACCCCAAT	CCACAGGCATTGCACAGGTA
Col4a4	NM_007735.2	AGCAAGCGGATGACAAAGA	AGCCAGAAGCCCAATAGATTAC
Id1	NM_010495.3	CGCTCAGCACCCTGAACGGC	TCCGGTGGCTGCGGTAGTGT
Acvlr1/ALK1	NM_009612.3	CCTCACGAGATGAGCAGTCC	GGCGATGAAGCCTAGGATGTT
Acvr1/ALK2	NM_007394.3	GTCATGGTTCAGGGAGACGG	CCAGAGTAGTGAGCTGAAGGT
Tgfbr1/ALK5	NM_009370.3	TTGCAGACTTGGGACTTGCT	GGGCCATGTACCTTTTAGTGC
Eng	NM_001146348.1	GCCAAAGTGTGGCAATCAGG	TGGTCGTCAGTGTCTTCAGC
Bmpr2	NM_007561.4	TGGCAGTGAGGTCACTCAAG	TTGCGTTCATTCTGCATAGC
Acvr2a/ActRIIA	NM_007396.4	AGCAAGGGGAAGATTTGGTT	GGTGCCTCTTTTCTCTGCAC
Cdh5/VE-cad	NM_009868.4	CTCCACAAAGCTCGGCCCTGG	AGGCCCAGGAAGGCTCCCAA
Smad1	NM_008539.4	CCGGGTCTCGTGCGTCC	TCTGCGGTTTGGAAAGGTAGAA
Smad5	NM_008541.3	TAATAAAGTTGCGGCGCGTG	AGAAAGTCCAAGTGGAGGCG
Smad9	NM_019483.5	CATGAGCTGAAGCCCTTGGA	CTTGGTACCAGCACTGGAGG
Vegfa	NM_001287056.1	GGCCTCCGAAACCATGAACTT	TGGGACCACTTGGCATGGTG
Smad6	NM_008542.3	CTGCGGGCCAGAATCACCGC	GCTCGGCTTGGTGGCATCCG
Smad7	NM_001042660.1	CAAACCAACTGCAGGCTGTC	CCCCAGGGGCCAGATAATTC
Flt1/VEGFR1	NM_010228.4	GTGTCTATAGGTGCCGAGCC	GCGTGATCAGCTCCAGGTTT
Kdr/VEGFR2	NM_010612.3	CTAGGCGCCTGCACCAAGCC	CTTGCCCTGGCGGAAGCGTG
Flt4/VEGFR3	NM_008029.3	GTGGTCCTTCGGCGTGCTGC	GTGGCGTATGGCAGGAGTGGC

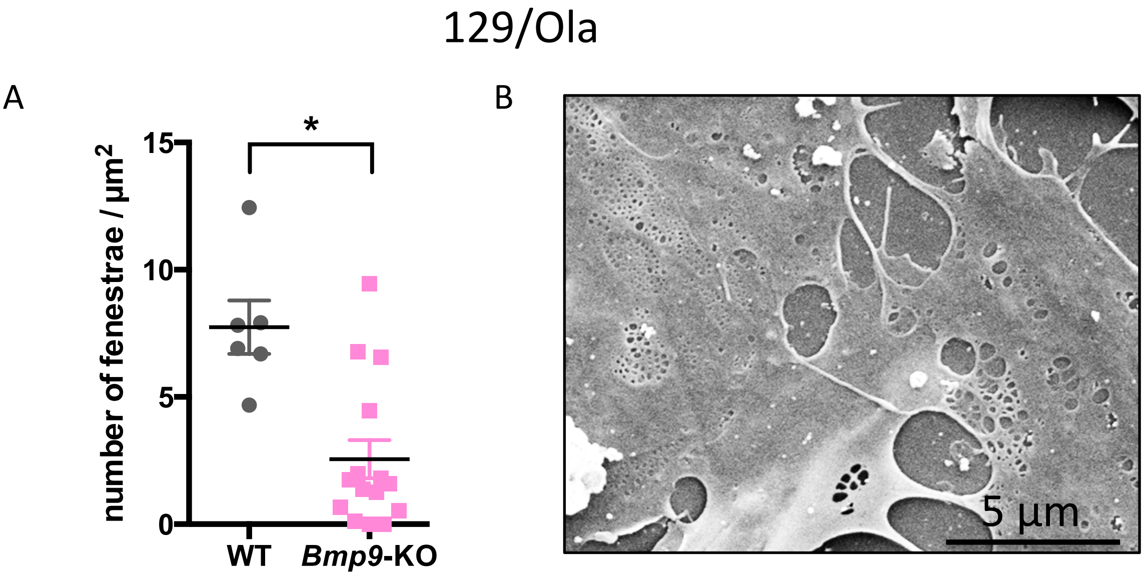

Supplementary Figure 1

Supplementary Figure 1. Loss of LSEC fenestration in *Bmp9*-KO mice in the 129/Ola background

A. Quantification of fenestrae by Scanning Electron Microscopy (SEM) of WT mice in the 129/Ola genetic background 6 hours after freshly isolated LSEC plating. Results were analyzed using Mann-Whitney statistical analysis * $p<0.05$.

B. Representative picture of SEM on freshly isolated LSEC from 129/Ola WT mice (females of 26 weeks old) 6 hours after plating.
